# Intestinal Bacterial Overgrowth in Severe Traumatic Brain Injury Is Associated with Late In-Hospital Mortality

**DOI:** 10.3390/jcm15207896

**Published:** 2026-10-13

**Authors:** Oleksandr Oliynyk, Wojciech Barg, Oleksandr Yashan, Konstiantyn Krenov, Yury Kovalchuk, Svitlana Yaroslavska, Marta Rorat

**Affiliations:** 1 Department of Anesthesiology and Intensive Care, Bogomolets National Medical University, 01601 Kyiv, Ukraine; alexanderoliynyk8@gmail.com (O.O.);; 2 Department of Health Promotion, Faculty of Physical Culture Sciences, Medical College, University of Rzeszów, 35-310 Rzeszów, Poland; wbarg@ur.edu.pl; 3 Department of Otolaryngology and Neurosurgery, Ternopil National Medical University, 46000 Ternopil, Ukraine; 4 Department of Surgery with a Course in the Basics of Dentistry, Faculty of Postgraduate Education, Vinnytsia National Pirogov Memorial Medical University, 29000 Khmelnytskyi, Ukraine; 5 Department of Social Sciences and Infectious Diseases, Wroclaw University of Science and Technology, 50-370 Wroclaw, Poland

**Keywords:** traumatic brain injury, intestinal bacterial overgrowth, dysbiosis, gut–brain axis, sepsis, mortality, intensive care

## Abstract

**Background**: Severe traumatic brain injury (TBI) is associated with high mortality. There is increasing evidence suggesting that intestinal dysbiosis may influence clinical outcomes; however, its prognostic significance in patients with severe TBI remains poorly defined. **Methods**: This retrospective cohort study included 235 patients with severe TBI (Glasgow Coma Scale [GCS] score ≤ 8) admitted to the intensive care units of three participating hospitals between September 2023 and December 2024. Quantitative microbiological analysis of jejunal aspirates was performed at admission and repeated on days 12–14 of the ICU stay. Small intestinal bacterial overgrowth (SIBO) was defined as a bacterial load of ≥10^5^ colony-forming units per milliliter (CFU/mL). Multivariable logistic regression and receiver operating characteristic (ROC) curve analyses were performed. **Results**: At admission, SIBO was uncommon (3.8%) but increased to 27.9% among patients who underwent repeated assessment. Within the day-14 landmark cohort (*n* = 179), post-day-14 mortality was higher in patients with SIBO than in those without (34.0% vs. 23.3%). In multivariable logistic regression analysis, SIBO was independently associated with post-day-14 in-hospital mortality after adjustment for age, body mass index, GCS score, PaO_2_/FiO_2_ ratio, and blood glucose (OR 2.83, 95% CI 1.06–7.58; *p* = 0.039). The ROC model demonstrated good discriminative performance, with an apparent area under the curve (AUC) of 0.846 and an optimism-corrected AUC of 0.823 after bootstrap validation. **Conclusions**: In patients with severe TBI who survived and remained hospitalized through the day-14 landmark, SIBO detected at days 12–14 was independently associated with increased subsequent in-hospital mortality. However, its incremental contribution to discrimination beyond established clinical predictors was modest. Therefore, late SIBO should be interpreted primarily as a dynamic marker associated with prolonged critical illness and adverse outcome rather than as a demonstrated cause of mortality. Prospective validation is required.

## 1. Introduction

Traumatic brain injury (TBI) is one of the leading causes of death and disability worldwide, with an estimated annual incidence exceeding 50 million cases [1,2]. Severe TBI is associated with high in-hospital mortality and long-term neurological impairment despite advances in neurosurgical techniques and intensive care management [3].

The clinical course and outcome of severe TBI are determined not only by the initial mechanical insult but also by a complex cascade of secondary injury mechanisms. These include cerebral hypoxia, systemic inflammation, metabolic disturbances, and multiple organ failure [4]. There is increasing evidence to suggest that systemic factors are of crucial importance in modulating neurological recovery, highlighting the importance of a holistic approach in the management of patients with severe TBI [5].

Traditional prognostic models, such as IMPACT and CRASH, incorporate demographic, neurological, and physiological variables, including age, Glasgow Coma Scale (GCS) score, and indices of oxygenation and metabolism [6]. Although these models provide valuable risk stratification, their predictive accuracy remains limited, particularly in the later phases of critical illness. These limitations suggest that dynamic biomarkers reflecting evolving systemic pathophysiology may provide additional prognostic value beyond admission characteristics.

In recent years, there has been an increasing focus on the role of the gastrointestinal tract in critical illness. The concept of the “gut as the motor of critical illness” proposes that intestinal barrier dysfunction, microbial imbalance, and bacterial translocation contribute to systemic inflammatory response, sepsis, and multiple organ failure [7]. In critically ill patients, profound alterations in gut microbiota composition can occur within days of intensive care unit (ICU) admission and are associated with unfavorable clinical outcomes [8,9].

Small intestinal bacterial overgrowth (SIBO), defined as an abnormal increase in bacterial load in the small intestine (≥10^5^ colony-forming units per milliliter [CFU/mL]), is one of the key manifestations of intestinal dysbiosis [10]. Although SIBO has been studied extensively in gastroenterology, its clinical relevance in critically ill populations remains incompletely understood.

Patients with severe TBI are particularly susceptible to gastrointestinal dysfunction due to impaired autonomic regulation, reduced intestinal motility, altered splanchnic perfusion, and neurohumoral dysregulation. In addition, intensive care interventions—including mechanical ventilation, sedation, antibiotic therapy, and proton pump inhibitors—further disrupt intestinal homeostasis [11,12]. These factors create a permissive environment for the development of SIBO. Notably, intestinal dysbiosis in critical illness is not merely a local phenomenon. Disruption of the intestinal barrier may lead to translocation of bacteria and microbial products into the systemic circulation. This may result in amplified systemic inflammation and, consequently, in the development of sepsis and multiple organ dysfunction. In this context, SIBO may represent both a potential contributor to disease progression and a marker of systemic physiological deterioration.

Despite these considerations, the prognostic significance of SIBO in patients with severe TBI has not been adequately investigated. In particular, it is unclear whether SIBO is merely a consequence of long-term intensive therapy or whether it reflects clinically relevant pathophysiological processes associated with an increased risk of death. We hypothesized that SIBO developing during prolonged intensive care is associated with increased in-hospital mortality in patients with severe TBI.

## 2. Study Endpoint

This study aimed to evaluate the prognostic value of small intestinal bacterial overgrowth assessed both at admission and on days 12–14 of ICU treatment. The primary endpoint of the landmark analysis was post-day-14 in-hospital mortality, defined as death occurring during the index hospitalization after the day-14 landmark. This analysis included patients who were alive and remained hospitalized at the time of repeat microbiological assessment on days 12–14.

## 3. Materials and Methods

### 3.1. Study Design and Treatment Protocol

This retrospective cohort study included 235 consecutive adult patients with severe traumatic brain injury (TBI) and a Glasgow Coma Scale (GCS) score ≤ 8 who were admitted to the intensive care units (ICUs) of Ternopil Emergency Hospital, Khmelnytskyi Regional Hospital, and Kyiv Regional Hospital from 1 September 2023 to 29 December 2024.

Exclusion criteria included acute myocardial infarction, cardiogenic pulmonary edema, decompensated chronic obstructive pulmonary disease, pulmonary embolism, chronic inflammatory bowel diseases, malignancy, immunosuppression, diabetic ketoacidosis, liver cirrhosis, and end-stage renal failure.

All patients were managed according to the most recent intensive care standards for severe traumatic brain injury (TBI), including intracranial pressure control, respiratory support, sedation, hemodynamic stabilization, prevention of secondary brain injury, and early enteral nutrition. To minimize treatment-related variability, the participating ICUs followed harmonized neurocritical care protocols based on contemporary international guidelines. Core treatment principles, including intracranial pressure management, ventilatory support, sedation and analgesia, hemodynamic optimization, nutritional support, thromboprophylaxis, and stress-ulcer prophylaxis, were applied consistently across the centers [13].

To control intracranial pressure (ICP), a multimodal neuroprotective strategy was applied. Hyperosmolar therapy represented the cornerstone of ICP management. Depending on the clinical response, hemodynamic status, renal function, and serum sodium levels, either 20% mannitol or 3% hypertonic saline was administered. Mannitol was given as an intravenous bolus at a loading dose of 0.5–1 g/kg, followed by repeated doses of 0.25–0.5 g/kg as needed. Alternatively, 3% hypertonic saline was administered as a bolus at a dose of 2–4 mL/kg, followed by a low-rate continuous infusion titrated according to clinical response. The choice of hyperosmolar agent was individualized, considering the risk of hypovolemia, renal dysfunction, and hypernatremia. Serum osmolality, sodium concentration, and other electrolytes were regularly monitored, and therapy was adjusted to maintain safe physiological ranges.

All patients were positioned with head elevation of 30–45° to facilitate cerebral venous outflow and reduce intracranial pressure. Care was taken to maintain a neutral head and neck position to avoid jugular venous compression. Body temperature was carefully monitored and controlled to avoid hyperthermia, which may exacerbate secondary brain injury. Hemodynamic management was directed toward maintaining adequate cerebral perfusion pressure and preventing secondary cerebral ischemia. Fluid therapy and vasopressor support were administered when necessary to ensure sufficient systemic arterial pressure. Norepinephrine was used as the first-line vasopressor when vasopressor therapy was required.

As all the patients required invasive mechanical ventilation, respiratory parameters reflected values obtained under controlled ventilatory support rather than spontaneous respiratory physiology. Ventilator settings were continuously adjusted to maintain adequate pH and avoid excessive deviations in PaO_2_ and PaCO_2_. Mechanical ventilation was delivered using lung-protective strategies. Ventilatory management aimed to maintain PaCO_2_ within the range of 35–40 mmHg to avoid both hypercapnia-induced increases in ICP and excessive hypocapnia associated with reduced cerebral perfusion. Adequate oxygenation was ensured to prevent hypoxic secondary brain injury. Neuroprotective sedation was managed using propofol as the primary sedative agent (loading dose 1–2 mg/kg; maintenance infusion 1–3 mg/kg/h). Sedation was titrated individually according to hemodynamic stability, ICP control, and the requirements of neurological monitoring. When indicated, sedation was combined with opioid analgesics to achieve adequate analgesia and ventilator synchrony.

Pharmacological thromboprophylaxis with low-molecular-weight heparin was administered unless contraindicated by active bleeding or neurosurgical considerations. The jejunal aspirates were collected during routine, clinically indicated upper gastrointestinal endoscopy performed for diagnostic assessment of stress-related gastroduodenal lesions. Stress ulcer prophylaxis was routinely provided using proton pump inhibitors. Blood glucose levels were monitored regularly and managed according to institutional intensive care protocols. If wound infection was suspected, microbiological cultures of wound exudate were obtained as part of routine clinical care to identify causative pathogens and guide antimicrobial therapy. Antibacterial therapy was initiated when clinically indicated and subsequently adjusted according to microbiological findings and antimicrobial stewardship principles. Antifungal prophylaxis was administered according to institutional protocols.

Because antimicrobial therapy was prescribed according to individual clinical indications, antibiotic exposure was not identical across patients. Detailed longitudinal data on cumulative antibiotic exposure, including treatment duration and dose, were not sufficiently standardized in the retrospective database to permit their reliable inclusion as covariates in the current analysis.

### 3.2. Intestinal Sampling and Microbiological Analysis

Early enteral nutrition was initiated within the first 24 h after ICU admission. This approach was intended to preserve intestinal barrier function, reduce the risk of bacterial translocation, and support metabolic demands during critical illness. During tube placement, a sterile single-use aspiration catheter (5–6 Fr; compatible with the working channel of the endoscope) was positioned distally to the ligament of Treitz into the proximal jejunum. After accurate positioning, aspiration of small intestinal contents was performed under sterile conditions. To minimize contamination from gastric and oropharyngeal flora, the initial small volume of aspirated fluid was discarded. Subsequently, 3–5 mL of jejunal aspirate was collected into sterile microbiological transport containers and immediately transferred for quantitative microbiological analysis. Samples were obtained at two predefined time points: at admission (day 1) and on days 12–14 of ICU stay. Repeated sampling was performed at the predefined follow-up time point (days 12–14) in patients who remained alive and hospitalized at that time, irrespective of the presence of gastrointestinal symptoms or complications. The repeat assessment was therefore not restricted to patients selected because of clinical deterioration or gastrointestinal complications. The 12–14-day interval was selected to evaluate SIBO developing during prolonged intensive care.

Microbiological analyses were performed in the microbiology laboratories of the participating state hospitals by trained clinical microbiology personnel in accordance with national Ukrainian microbiological laboratory requirements and standardized operating procedures implemented in the respective laboratories. Quantitative bacteriological analysis was performed using standard culture-based techniques. Samples were inoculated onto 5% sheep blood agar and Endo agar plates and incubated under standard laboratory conditions. Quantitative bacterial load was determined by colony counting and expressed as colony-forming units per milliliter (CFU/mL). When required, serial dilutions were performed to improve the accuracy of bacterial quantification. Bacterial isolates were identified using the VITEK^®^ 2 Compact automated identification system (bioMérieux, Marcy-l’Étoile, France). Antimicrobial susceptibility testing was performed according to institutional laboratory standards when clinically indicated. Laboratory procedures, including culture handling and quality-control measures, were performed according to applicable national requirements and local standardized procedures. Quantitative bacterial load (CFU/mL) was recorded for each sample and used as the primary microbiological variable in subsequent statistical analyses.

### 3.3. Definitions

Small intestinal bacterial overgrowth (SIBO) was defined as a bacterial load ≥ 10^5^ CFU/mL in jejunal aspirates. This threshold was selected because samples were obtained from the proximal jejunum distal to the ligament of Treitz, and ≥10^5^ CFU/mL represents the traditional microbiological criterion for SIBO based on quantitative jejunal aspirate culture. Although current recommendations have proposed a lower threshold of ≥10^3^ CFU/mL, particularly for duodenal aspirates, the optimal cut-off remains dependent on the sampling site and is not fully standardized. In this study, the higher threshold was used as a conservative criterion for substantial bacterial overgrowth.

**Figure 1:**
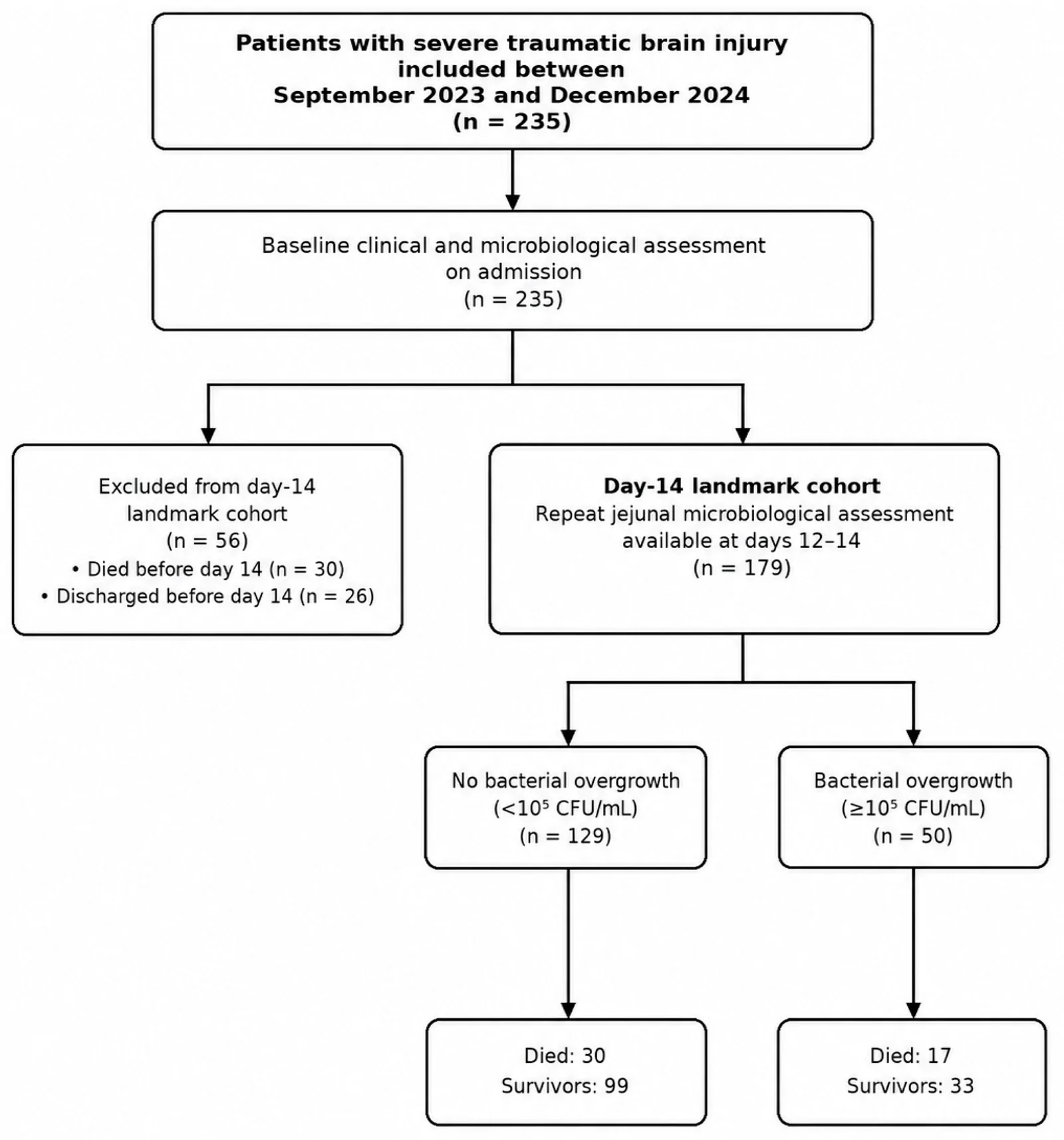
STROBE flow diagram of patient inclusion, landmark cohort selection, and classification according to small intestinal bacterial overgrowth (SIBO) at days 12–14. Patients who died or were discharged before day 14 were excluded from the landmark cohort to avoid mortality time bias.

The landmark cohort was defined as a set of patients who were suitable for the intestinal microbiological assessment on days 12–14. From a total of 235 patients who entered the study, 179 had the follow-up intestinal microbiological test performed and constituted the predefined post-day-14 landmark cohort. By restricting the analysis to patients who were alive and under observation at the time of repeated sampling, the landmark design reduced the risk of mortality time bias. The study flowchart is shown in Figure 1.

Sepsis was defined according to the Sepsis-3 criteria as life-threatening organ dysfunction caused by a dysregulated host response to infection. In clinical practice, sepsis was identified by documented or suspected infection accompanied by an increase in the Sequential Organ Failure Assessment (SOFA) score by ≥2 points from baseline.

Marshall CT classification was used as a standardized radiological measure of TBI severity. The classification is based on computed tomography findings, including diffuse injury, intracranial mass lesions, midline shift, and basal cistern status, with higher scores reflecting more severe structural brain injury [14].

For the landmark analysis, post-day-14 in-hospital mortality was defined as death occurring during the index hospitalization after the day-14 landmark.

### 3.4. Statistical Analysis

Statistical analysis was performed using MedCalc^®^ Statistical Software (version 20.009) and Python (version 3.10; statsmodels 0.14; scikit-learn 1.3).

Continuous variables were expressed as median and interquartile range (IQR) and compared using the Mann–Whitney U test. Categorical variables were compared using the chi-square test or Fisher’s exact test, as appropriate.

Variables associated with mortality in univariable analyses, together with clinically relevant covariates, were considered for inclusion in multivariable logistic regression models. To reduce the risk of model instability and overfitting, a parsimonious modeling strategy was applied.

The primary multivariable analysis was performed within the predefined day-14 landmark cohort and evaluated predictors of post-day-14 in-hospital mortality. Adjusted odds ratios (ORs) with 95% confidence intervals (CIs) were calculated.

Model discrimination was assessed using receiver operating characteristic (ROC) curve analysis and quantified by the area under the curve (AUC). Internal validation of the final multivariable logistic regression model was performed using 1000 bootstrap resamples. In each bootstrap sample, the model was refitted, and its discriminative performance was compared with the performance in the original landmark cohort. The average optimism was then subtracted from the apparent AUC to obtain the optimism-corrected AUC.

To assess the incremental prognostic value of SIBO, the clinical model including age, BMI, GCS score, PaO_2_/FiO_2_ ratio, and blood glucose was compared with the same model additionally including SIBO at days 12–14. Discrimination was compared using the DeLong test for correlated ROC curves. Overall predictive performance was assessed using the Brier score. The overall fit of the nested models was compared using the likelihood-ratio test.

Because the number of outcome events was relatively limited in relation to the number of candidate predictors, the possibility of residual overfitting cannot be excluded despite the use of a parsimonious modeling strategy and bootstrap validation. All statistical tests were two-sided, and *p*-values < 0.05 were considered statistically significant.

## 4. Results

Baseline demographic and clinical characteristics of the total study population and the landmark cohort are presented in Table 1.

At admission, SIBO was identified in 9 of 235 patients (3.8%), whereas the majority of patients (96.2%) presented no evidence of clinically significant bacterial overgrowth. As demonstrated in Table 2, the prevalence of SIBO increased more than sevenfold during the ICU stay.

In the overall study population, in-hospital mortality was observed in 80 of 235 patients (34.0%), and in the landmark cohort, post-day-14 in-hospital mortality corresponded to 47 of 179 patients (26.3%). Notably, patients with SIBO at days 12–14 experienced a higher rate of in-hospital mortality than patients without SIBO (Table 3).

Sepsis occurred more frequently among non-survivors than among survivors (Table 4).

A multivariable logistic regression model was constructed to identify independent predictors of post-day-14 in-hospital mortality (Table 5). Age remained independently associated with a higher risk of mortality. Higher body mass index (BMI) was also independently associated with increased mortality risk after adjustment for the remaining covariates.

Higher Glasgow Coma Scale (GCS) scores demonstrated an independent protective association with mortality, indicating that patients with better neurological status had significantly lower odds of death. Similarly, higher PaO_2_/FiO_2_ ratios were independently associated with improved survival, suggesting that respiratory dysfunction remained an important determinant of adverse outcome. In contrast, blood glucose levels were not independently associated with mortality after adjustment.

Among microbiological variables, SIBO at days 12–14 (≥10^5^ CFU/mL) remained independently associated with post-day-14 in-hospital mortality after adjustment for major clinical severity markers (OR 2.83, 95% CI 1.06–7.58, *p* = 0.039).

In an exploratory sensitivity analysis, sepsis occurring at any time during hospitalization was added to the primary multivariable model. The association between SIBO at days 12–14 and subsequent in-hospital mortality was attenuated and was no longer statistically significant (OR 0.33, 95% CI 0.08–1.32; *p* = 0.118). However, only two patients had developed sepsis by the day 12–14 microbiological assessment, whereas the remaining sepsis events subsequently occurred. Thus, this analysis involved a predominantly post-exposure variable and was considered exploratory.

**Figure 2:**
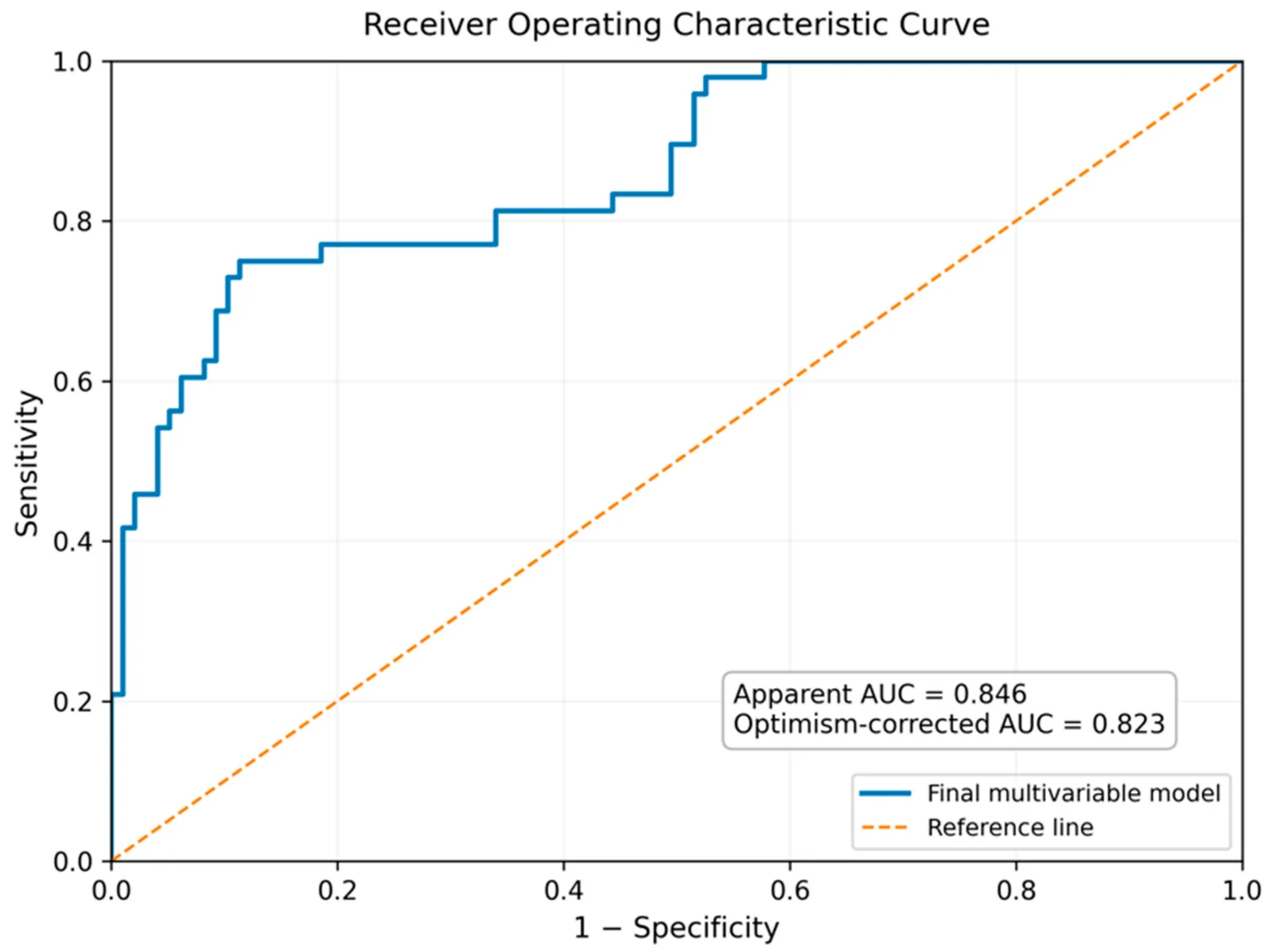
Receiver operating characteristic (ROC) curve of the multivariable logistic regression model for the prediction of post-day-14 in-hospital mortality in the day-14 landmark cohort. The model included age, body mass index (BMI), Glasgow Coma Scale (GCS) score, PaO_2_/FiO_2_ ratio, blood glucose, and small intestinal bacterial overgrowth (≥10^5^ CFU/mL) at days 12–14.

Receiver operating characteristic (ROC) analysis was performed to evaluate the discriminative performance of the multivariable logistic regression model for the prediction of post-day-14 in-hospital mortality in the predefined day-14 landmark cohort (Figure 2). The final model included age, body mass index (BMI), Glasgow Coma Scale (GCS) score, PaO_2_/FiO_2_ ratio, blood glucose level, and SIBO at days 12–14 (≥10^5^ CFU/mL). The model demonstrated good discriminative performance, with an apparent AUC of 0.846 and an optimism-corrected AUC of 0.823 after bootstrap validation.

Incremental prognostic value of SIBO: The clinical model without SIBO yielded an AUC of 0.839. Addition of SIBO at days 12–14 increased the AUC to 0.846 (ΔAUC = 0.007; DeLong *p* = 0.586). The Brier score decreased slightly from 0.129 to 0.127. Although the improvement in discrimination was small and not statistically significant, the addition of SIBO improved overall model fit in the likelihood-ratio test (χ^2^ = 4.34, *p* = 0.037).

## 5. Discussion

A key finding of this study is a strong association between small intestinal bacterial overgrowth (SIBO) assessed at days 12–14 of ICU treatment and post-day-14 in-hospital mortality in patients with severe traumatic brain injury (TBI). Patients with SIBO had approximately 2.8-fold higher odds of subsequent in-hospital death compared with those without SIBO (OR 2.83, 95% CI 1.06–7.58), although the wide confidence interval indicates substantial uncertainty in the magnitude of this association. These findings indicate an association between SIBO and subsequent mortality after accounting for mortality time bias through the landmark design. To our knowledge, there are no studies directly evaluating the prognostic significance of SIBO in patients with severe TBI. Previous research has primarily focused on gut microbiota dysbiosis and its potential role in neuroinflammation, systemic immune dysfunction, and the gut–brain axis rather than on quantitative assessment of small intestinal bacterial overgrowth or its association with mortality [11,12,15].

While SIBO in our patients was uncommon at admission, it became substantially more frequent during the second week of hospitalization. These observations suggest that SIBO in patients with severe TBI is predominantly a dynamic phenomenon developing during intensive care rather than a pre-existing condition present at admission. Notably, baseline microbiological findings were not associated with the outcome, whereas bacterial overgrowth detected at days 12–14 was associated with subsequent mortality. This temporal pattern is consistent with previous studies showing rapid microbiota disruption in critically ill patients, influenced by mechanical ventilation, reduced enteral feeding, antibiotic exposure, and impaired gut motility [15]. Collectively, these observations support the concept that the gut may contribute to systemic inflammation during critical illness. The “gut-origin of sepsis” hypothesis proposes that intestinal barrier dysfunction, bacterial translocation, and microbiota alterations contribute to systemic inflammatory response and multiple organ failure [7].

Beyond the gut–brain axis, these findings may also be considered within the broader framework of gut–systemic and inter-organ crosstalk. Disruption of intestinal homeostasis and barrier integrity may facilitate bacterial translocation and systemic inflammatory signaling, potentially contributing to sepsis and secondary dysfunction of distant organs, including the kidneys and liver. Similar concepts of pathological inter-organ communication mediated by inflammatory and metabolic signaling have been described for other organ systems, including the liver–kidney axis [16]. Although such evidence does not directly demonstrate an analogous mechanism in TBI, it provides a useful conceptual framework for interpreting intestinal dysbiosis as part of a broader systemic response to critical illness. In this study, these mechanisms remain hypothetical and cannot be established from the associations observed.

The microbiological definition of SIBO also warrants consideration. The ≥10^5^ CFU/mL threshold used in this study represents the traditional criterion for quantitative culture of jejunal aspirates, whereas lower thresholds, particularly ≥10^3^ CFU/mL, have been proposed in contemporary recommendations [10]. In critically ill patients, interpretation of intestinal bacterial counts may be further complicated by impaired gastrointestinal motility, antibiotic exposure, proton pump inhibitor therapy, enteral nutrition, and other ICU-related interventions that substantially alter intestinal microbial ecology. Therefore, we used the more conservative ≥10^5^ CFU/mL threshold to identify substantial bacterial overgrowth. However, this approach may have failed to identify lower-grade bacterial overgrowth, and the optimal diagnostic threshold for SIBO in critically ill patients remains to be established prospectively [10].

There are several clinical factors that undoubtedly affect mortality in severe TBI. Consistent with previous large multicenter studies, age and impaired neurological status at admission were significant predictors of mortality. Age remains one of the most robust prognostic factors in TBI, with a near-linear association with mortality risk [1]. Similarly, the Glasgow Coma Scale (GCS) score is a cornerstone of prognostic assessment and is incorporated into widely used models such as IMPACT and CRASH [6]. In addition, respiratory dysfunction, reflected by a reduced PaO_2_/FiO_2_ ratio, was independently associated with mortality. This finding aligns with the concept of secondary brain injury, where systemic hypoxia contributes to ongoing neuronal damage and worsens outcomes [4].

In our study, SIBO detected during the ICU stay was associated with subsequent mortality in the primary multivariable analysis. Because sepsis may represent an intermediate event occurring along the pathway between intestinal bacterial overgrowth and mortality, it was not included in the primary model. The sensitivity analysis including sepsis requires caution because of temporal misalignment between the variables. SIBO was assessed at the day 12–14 landmark, whereas only two patients had developed sepsis by that time, and most sepsis events occurred later during the ICU course. Consequently, sepsis occurring at any time during hospitalization cannot be regarded as a baseline confounder of the association between day 12–14 SIBO and subsequent mortality. Its inclusion in a conventional logistic regression model may constitute overadjustment if sepsis represents an intermediate event between intestinal dysbiosis and adverse outcome. Alternatively, both SIBO and subsequent sepsis may reflect the severity and duration of critical illness. Therefore, the attenuation of the SIBO coefficient after adjustment for sepsis should not be interpreted as evidence either for or against mediation. Establishing these temporal relationships would require prospective time-dependent analyses.

Only patients who underwent repeat microbiological assessment on days 12–14 were included in the landmark cohort and were eligible for subsequent analyses. Thus, lower mortality observed within the landmark cohort as compared with the overall study population is an expected consequence of the landmark design. Although these findings were derived from an unadjusted comparison, the observed difference in mortality between groups suggested a potential association between late SIBO and adverse clinical outcomes. This hypothesis was subsequently evaluated using multivariable logistic regression models adjusted for established clinical severity markers. Generally, these descriptive findings support the concept that SIBO developing during a prolonged ICU stay may be associated with an increased risk of mortality in patients with severe TBI.

The final multivariable model demonstrated good overall discrimination, with limited optimism after bootstrap validation. However, the incremental prognostic contribution of SIBO beyond established clinical variables was modest. The addition of SIBO only increased the AUC from 0.839 to 0.846, and this improvement in discrimination was not statistically significant according to the DeLong test (*p* = 0.586). Similarly, the Brier score showed only a small improvement. Nevertheless, the likelihood-ratio test indicated a statistically significant improvement in overall model fit after inclusion of SIBO (*p* = 0.037). These findings suggest that although SIBO was independently associated with subsequent mortality, its additional contribution to individual risk discrimination beyond established clinical predictors was limited.

Residual confounding related to unmeasured markers of disease severity and treatment-related effects cannot be excluded. Sepsis should be taken into account in particular. It is well known that sepsis is associated with profound alterations in gut microbiota, including loss of commensal diversity and overgrowth of opportunistic pathogens [8,9]. At the same time, intestinal dysbiosis itself may promote bacterial translocation and contribute to the development of sepsis [17]. Thus, one can presume that a bidirectional relationship exists, where dysbiosis promotes sepsis, and sepsis further exacerbates gut barrier dysfunction and microbial imbalance.

The temporal increase in intestinal bacterial overgrowth during the ICU stay suggests that it may develop as a consequence of prolonged critical illness and its associated interventions. However, the relationship may be bidirectional, as bacterial overgrowth could potentially contribute to intestinal barrier dysfunction, bacterial translocation, systemic inflammation, and infectious complications. Given the observational design of this study, causality cannot be established, and late intestinal bacterial overgrowth should therefore be interpreted primarily as a dynamic marker associated with disease progression and adverse outcomes rather than as a demonstrated cause of mortality.

Information on the causative pathogens would be valuable. However, this retrospective study included patients treated in three different hospitals, and the predominant bacterial flora differed considerably between the participating centers. Moreover, many patients had polymicrobial infections, and the spectrum of isolated microorganisms often changed during the ICU stay. Therefore, the manuscript is limited to reporting the frequency of sepsis without presenting pathogen-specific analyses.

A clinically relevant observation in our cohort was that sepsis occurred more than three times as frequently among non-survivors as among survivors (Table 4). This finding, together with the observed association between late SIBO and subsequent mortality, supports the hypothesis that intestinal dysbiosis may reflect broader systemic physiological disturbances during prolonged critical illness. However, the temporal and causal relationships between SIBO, sepsis, and adverse outcomes cannot be established from the current observational data. SIBO detected at days 12–14 should therefore be interpreted primarily as a dynamic marker associated with systemic deterioration and adverse outcomes rather than as a demonstrated cause of mortality. Moreover, given its modest incremental prognostic contribution beyond established clinical predictors, its utility for individual risk prediction remains uncertain and requires prospective validation.

These results raise the question of whether intestinal dysbiosis should be actively targeted in critically ill patients. Potential strategies may include optimization of enteral nutrition, modulation of gut motility, and microbiota-directed therapies such as probiotics or selective decontamination approaches [18]. However, the efficacy and safety of such interventions in patients with severe TBI remain uncertain and require prospective investigation.

An important methodological strength of this study is the use of a landmark analysis at day 14, which minimizes potential mortality time bias by ensuring that microbiological exposure is only assessed in patients who are alive and at risk at the same time point [19]. The strength of the association between SIBO and subsequent in-hospital mortality should be interpreted cautiously. Although SIBO remained independently associated with mortality in the primary multivariable model (OR 2.83, 95% CI 1.06–7.58; *p* = 0.039), the wide confidence interval indicates substantial uncertainty in the magnitude of the effect. In addition, the limited number of outcome events relative to the number of predictors increases the risk of model overfitting and potential inflation of effect estimates. External validation in independent cohorts is therefore required.

The study has several limitations that should be considered when interpreting the findings. The retrospective design precludes causal inference and limits the ability to fully account for time-dependent confounding. Effects estimated from retrospective analyses, especially within selected subgroups, may be inflated and sensitive to sample size and event distribution.

Residual confounding related to unmeasured indicators of disease severity and ICU course remains possible. In particular, variables such as duration of mechanical ventilation, sepsis, hemodynamic instability, and treatment-related factors were not comprehensively investigated. Because this was a retrospective multicenter study, detailed standardized records of aerobic and anaerobic incubation conditions and formal inter-laboratory reproducibility testing were not available retrospectively for all participating centers.

Treatment-related factors may have influenced the intestinal microbiota [20,21]. Proton pump inhibitors and other ICU-related therapies were administered according to institutional protocols, whereas antimicrobial therapy was prescribed according to individual clinical indications and was therefore not identical across patients. Detailed longitudinal data on cumulative antimicrobial exposure, including treatment duration and dose, were not sufficiently standardized in the retrospective database to permit adjustment for these variables. Consequently, residual confounding related to differences in antimicrobial exposure cannot be excluded.

The day-14 landmark design necessarily restricted the follow-up analysis to patients who survived and remained hospitalized until the repeat microbiological assessment. Although this approach reduces mortality-time bias, it does not eliminate potential selection bias. In particular, inclusion in the landmark cohort corresponded to patients with a sufficiently prolonged hospital course to undergo repeat assessment, while patients who died or were discharged before day 14 were excluded. Repeat sampling was performed at the predefined follow-up time point irrespective of gastrointestinal symptoms or clinical deterioration, thereby reducing the potential for indication-related selection; nevertheless, survivor and length-of-stay selection cannot be excluded. Accordingly, the results of the landmark analysis apply primarily to patients with severe TBI who survive and remain hospitalized through the second week of intensive care and should not be generalized to patients with early mortality or early discharge.

Finally, the study was conducted in three centers within a limited regional healthcare setting, which may limit reproducibility and generalizability. External validation in independent, preferably prospective cohorts is required before definitive conclusions can be drawn.

## 6. Conclusions

SIBO detected at days 12–14 of ICU treatment was independently associated with increased post-day-14 in-hospital mortality in patients with severe TBI after adjustment for established clinical severity markers. However, its incremental contribution to discrimination beyond the clinical model was modest, indicating that SIBO should not be considered a stand-alone prognostic predictor.

The marked increase in SIBO prevalence during ICU stay suggests that it is predominantly a dynamic phenomenon developing during prolonged critical illness. Given the retrospective observational design, this study cannot determine whether SIBO contributes directly to subsequent deterioration or primarily reflects the severity and duration of critical illness and associated factors, including antimicrobial exposure, impaired intestinal motility, sepsis, and multiorgan dysfunction. Therefore, late SIBO should primarily be interpreted as a dynamic marker associated with disease progression and adverse outcomes rather than as a demonstrated cause of mortality.

Prospective multicenter studies with longitudinal assessment of intestinal microbiota and time-dependent clinical variables are required to validate these findings, clarify the temporal and mechanistic relationships between SIBO, sepsis, and adverse outcomes, and determine whether microbiota-targeted interventions may have clinical benefit.

## Figures and Tables

**Table 1:** Baseline demographic and clinical characteristics at ICU admission in the overall study population (*n* = 235) and in patients who subsequently constituted the predefined day-14 landmark cohort (*n* = 179).

Variable	Study Population (*n* = 235)	Landmark Cohort (*n* = 179)
Sex, male, *n* (%)	152 (64.7%)	116 (64.8%)
Age, years	47 (37–64)	46 (36–63)
Body mass index (BMI), kg/m^2^	31 (29–33)	31 (29–33)
Glasgow Coma Scale (GCS) score	7 (6–8)	7 (7–8)
PaO_2_/FiO_2_ ratio	356 (343–367)	356 (343–367)
Intracranial pressure (ICP), mmHg	14 (14–15)	14 (14–15)
PaCO_2_, mmHg	44 (43–45)	44 (43–45)
Blood glucose, mmol/L	9 (8–11)	9 (8–10)
Marshall CT classification	3 (2–3)	3 (2–3)

**Table 2:** Dynamics of small intestinal bacterial overgrowth (SIBO).

Time Point	Population	Without SIBO, *n*	with SIBO, *n* (%)
Day 1 (admission)	235	226	9 (3.8)
Days 12–14	179 *	129	50 (27.9)

* Assessment at days 12–14 was only available in patients who survived and remained hospitalized. Missing data at this time point correspond to patients who died or were discharged from the ICU before day 14.

**Table 3:** Post-day-14 mortality according to intestinal bacterial overgrowth status in the landmark cohort (*n* = 179). SIBO—small intestinal bacterial overgrowth ≥ 10^5^ CFU/mL.

Group	*n*	Deaths, *n*	Mortality (%)
Without SIBO	129	30	23.3
With SIBO	50	17	34

**Table 4:** Sepsis according to survival status in the overall cohort.

Group	*n*	Sepsis, *n* (%)
Survivors	155	25 (16.1%)
Non-survivors	80	53 (66.3%)

**Table 5:** Multivariable logistic regression analysis of post-day-14 in-hospital mortality in the landmark cohort (*n* = 179). SIBO—small intestinal bacterial overgrowth ≥ 10^5^ CFU/mL.

Variable	OR	95% CI	*p*-Value
Age (per 1 year)	1.06	1.02–1.09	0.001
BMI (kg/m^2^)	1.26	1.03–1.53	0.024
GCS (per 1 point)	0.60	0.40–0.91	0.015
PaO_2_/FiO_2_ (per 10-unit increase)	0.59	0.46–0.77	<0.001
Blood glucose (mmol/L)	1.04	0.83–1.31	0.720
SIBO	2.83	1.06–7.58	0.039

## Data Availability

The original contributions presented in this study are included in the article. Further inquiries can be directed to the corresponding author.

## Abbreviations

The following abbreviations are used in this manuscript:

AUC	Area under the curve
BMI	Body mass index
CFUs	Colony-forming units
CIs	Confidence intervals
GCS	Glasgow Coma Scale
ICU	Intensive care unit
ICP	Intracranial pressure
ORs	Odds ratios
ROC	Receiver operating characteristic
SIBO	Small intestinal bacterial overgrowth
SOFA	Sequential Organ Failure Assessment
TBI	Traumatic brain injury

## References

[1] Maas A.I.R., Menon D.K., Manley G.T., Abrams M., Åkerlund C., Andelic N., Aries M., Bashford T., Bell M.J., Bodien Y.G. (2022). Traumatic brain injury: Progress and challenges in prevention, clinical care, and research. Lancet Neurol..

[2] Rapp A., Kobeissi H., Fahim D.K. (2025). Updated Review of the Management of and Guidelines for Traumatic Brain Injury. J. Clin. Med..

[3] Khan T.A., Kamm S. (2023). State of the Globe: Traumatic Brain Injury and Infections: The Two-Hit Insult. J. Glob. Infect. Dis..

[4] Svedung Wettervik T., Beqiri E., Hånell A. (2023). Brain tissue oxygen monitoring in traumatic brain injury-part II: Isolated and combined insults in relation to outcome. Crit. Care.

[5] Gandasasmita N., Li J., Loane D.J., Semple B.D. (2024). Experimental Models of Hospital-Acquired Infections After Traumatic Brain Injury: Challenges and Opportunities. J. Neurotrauma.

[6] Dijkland S.A., Foks K.A., Polinder S., Dippel D.W.J., Maas A.I.R., Lingsma H.F., Steyerberg E.W. (2020). Prognosis in Moderate and Severe Traumatic Brain Injury: A Systematic Review of Contemporary Models and Validation Studies. J. Neurotrauma.

[7] Guha L., Agnihotri T.G., Jain A., Kumar H. (2023). Gut microbiota and traumatic central nervous system injuries: Insights into pathophysiology and therapeutic approaches. Life Sci..

[8] Kain T., Dionne J.C., Marshall J.C. (2024). Critical illness and the gut microbiome. Intensive Care Med..

[9] Zaborin A., Smith D., Garfield K., Quensen J., Shakhsheer B., Kade M., Tirrell M., Tiedje J., Gilbert J.A., Zaborina O. (2014). Membership and behavior of ultra-low-diversity pathogen communities present in the gut of humans during prolonged critical illness. mBio.

[10] Pimentel M., Saad R.J., Long M.D., Rao S.S.C. (2020). ACG Clinical Guideline: Small intestinal bacterial overgrowth. Am. J. Gastroenterol..

[11] Pan P., Song Y., Du X., Bai L., Hua X., Xiao Y., Yu X. (2019). Intestinal barrier dysfunction following traumatic brain injury. Neurol. Sci..

[12] Cannon A.R., Anderson L.J., Galicia K., Murray M.G., Kamran A.S., Li X., Gonzalez R.P., Choudhry M.A. (2023). Traumatic brain injury-induced inflammation and gastrointestinal motility dysfunction. Shock.

[13] Carney N., Totten A.M., O’Reilly C., Ullman J.S., Hawryluk G.W.J., Bell M.J., Bratton S.L., Chesnut R., Harris O.A., Kissoon N. (2017). Guidelines for the Management of Severe Traumatic Brain Injury, Fourth Edition. Neurosurgery.

[14] Edwards K.A., Day J.A., Yun J., Yun S., Dark H.E., Gabor L., Gill J.M. (2025). Association of Marshall CT Scores with GFAP, UCH-L1, Tau, NfL, and p-Tau231 After Traumatic Brain Injury. Int. J. Mol. Sci..

[15] Salameh T.J., Roth K., Schultz L., Ma Z., Bonavia A.S., Broach J.R., Hu B., Howrylak J.A. (2023). Gut microbiome dynamics and associations with mortality in critically ill patients. Gut Pathog..

[16] Bogdan R.G., Boicean A., Anderco P., Ichim C., Iliescu-Glaja M., Todor S.B., Leonte E., Bloanca V.A., Crainiceanu Z.P., Popa M.L. (2025). From Liver to Kidney: The Overlooked Burden of Nonalcoholic Fatty Liver Disease in Chronic Kidney Disease. J. Clin. Med..

[17] Jang S., Kim Y.J., Park J., Kim D., Kim T.H., Lee S., Kim D.J., Ryu C.M., Seo H.W. (2026). Muribaculaceae-enriched microbiota exacerbates TLR4-dependent Acinetobacter baumannii-induced hyperinflammatory sepsis. Nat. Commun..

[18] Holcomb M., Marshall A.G., Flinn H., Lozano-Cavazos M., Soriano S., Gomez-Pinilla F., Treangen T.J., Villapol S. (2025). Probiotic treatment induces sex-dependent neuroprotection and gut microbiome shifts after traumatic brain injury. J. Neuroinflamm..

[19] Anderson J.R., Cain K.C., Gelber R.D. (2008). Analysis of survival by tumor response and other comparisons of time-to-event by outcome variables. J. Clin. Oncol..

[20] Khurmatullina A.R., Andreev D.N., Kucheryavyy Y.A., Sokolov F.S., Beliy P.A., Zaborovskiy A.V., Maev I.V. (2025). The Duration of Proton Pump Inhibitor Therapy and the Risk of Small Intestinal Bacterial Overgrowth: A Systematic Review and Meta-Analysis. J. Clin. Med..

[21] Macke L., Schulz C., Koletzko L., Malfertheiner P. (2020). Systematic review: The effects of proton pump inhibitors on the microbiome of the digestive tract-evidence from next-generation sequencing studies. Aliment. Pharmacol. Ther..

